# Range-Aware Two-Stage Modeling for Feed Ratio Optimization in Fluoroelastomers: Mechanistic Pathways from NMR Structural Features to Macroscopic Properties

**DOI:** 10.3390/ma18194618

**Published:** 2025-10-06

**Authors:** Yaxian Liu, Yadong Wu, Zhoujun Lin, Lijuan Peng, Hongwei Fu

**Affiliations:** 1 College of Computer Science and Engineering, Sichuan University of Science and Engineering, Yibin 644002, China; liuyaxian25@gmail.com (Y.L.); fuhongwei04@163.com (H.F.); 2 Sichuan Engineering Research Center for Big Data Visual Analytics, Yibin 644002, China; 3 Key Laboratory of Higher Education of Sichuan Province for Enterprise Informationalization and Internet of Things, Yibin 644002, China; 4 Innovation Center for Chenguang High Performance Fluorine Material, Zigong 643200, China; linzhoujun@sinochem.com; 5 Organic Fluorine Material Key Laboratory of Sichuan Province, Zhonghao Chenguang Research Institute, Zigong 643200, China; 6 College of Computer Science and Engineering, Southwest University of Science and Technology, Mianyang 621010, China; plj@swust.edu.cn

**Keywords:** fluoroelastomer, range-aware modeling, mechanistic analysis, structure-property pathways, NMR characterization

## Abstract

This study developed the RATS (Range-Aware Two-Stage) modeling approach to establish mechanistic foundations for feed ratio optimization in fluoroelastomers. Using ^19^F NMR spectroscopic analysis, the approach decomposes complex composition–property relationships into sequential processes: monomer feed ratios to NMR-derived structural features, and structural features to properties, enabling mechanistic pathway analysis through quantifiable structural intermediates. Using 52 industrial datasets, RATS achieved an average R^2^ of 0.90 across four property predictions, representing a 0.14 improvement over direct modeling and a 28% reduction in prediction error. The approach identified 72 systematic transmission pathways, including promoting effects of PMVE-series structures (+0.220 influence strength) and inhibitory effects of VDF monomers (−0.219 influence strength), through quantified model parameter analysis. This methodology provides a practical analytical tool for mechanism-driven feed ratio optimization, facilitating the transition from empirical trial-and-error to systematic, data-guided fluoroelastomer formulation.

## 1. Introduction

Fluoroelastomer composition optimization faces a critical challenge: while monomer feed ratios significantly influence key properties (Mooney viscosity, tensile strength, compression set, and elongation at break), industrial data typically exhibit low variability characteristics (coefficient of variation < 15%) with 85% of samples clustering within narrow composition windows. This creates ‘high-stakes, low-signal’ optimization scenarios where subtle compositional changes (1–2%) significantly impact performance yet challenge conventional modeling approaches.

Fluoroelastomers, a class of specialty elastomeric materials renowned for their excellent comprehensive properties, have become indispensable in aerospace, automotive, and chemical industries owing to their superior high-temperature stability, corrosion resistance, oil resistance, and antioxidant properties [1,2]. The unique molecular architecture of fluoroelastomers, characterized by high-energy C-F bonds (485 kJ/mol) on the side chains, imparts exceptional chemical stability, allowing them to maintain performance integrity for extended durations at temperatures up to 250 °C and in highly corrosive environments [3,4]. With rapid industrial advancements and increasingly demanding application environments, stricter performance requirements are being placed on fluoroelastomers, particularly for the precise control of specific properties while balancing multiple performance attributes [5,6]. Moreover, increasing regulatory scrutiny of per- and polyfluoroalkyl substances (PFAS), including fluoropolymers used in fluoroelastomer production, has intensified the need for precise composition control and optimization strategies to ensure both performance excellence and regulatory compliance [7,8].

Fluoroelastomer composition optimization is a complex systems engineering challenge, encompassing multiple dimensions such as feed ratio design, vulcanization system optimization, and filler system configuration [9,10]. Among these, feed ratio design is pivotal, significantly dictating the polymer main chain’s chemical structure, functional group distribution, and intermolecular interactions. It is thus a key factor for targeted property regulation and composition optimization [11]. Modern fluoroelastomers typically utilize multicomponent copolymerization strategies, enabling targeted property control through the precise adjustment of different functional monomer feed ratios [12,13]. For instance, in widely used terpolymer fluoroelastomer systems, vinylidene fluoride (VDF) contributes to chain structure, tetrafluoroethylene (TFE) enhances chemical resistance and thermal stability, and perfluoromethyl vinyl ether (PMVE) modulates vulcanization and processing characteristics. The elastomeric properties arise from the synergistic combination of these monomers, where TFE and PMVE disrupt VDF crystallinity to achieve the desired elastic behavior [14,15,16]. Variations in these monomer feed ratios induce systematic changes in microstructural parameters like molecular chain sequence distribution, crystallinity, and glass transition temperature, which in turn exert complex synergistic effects on macroscopic properties such as Mooney viscosity, tensile strength, elongation at break, and hardness [9,17]. However, a quantitative understanding of these influence pathways through statistical modeling is currently lacking, hindering rational design optimization and systematic comprehension of fluoroelastomer formulations. This study focuses on elucidating the quantitative influence pathways from monomer composition changes to property variations through microstructural mediators.

Traditional fluoroelastomer composition optimization primarily relies on trial-and-error, which, despite its widespread application, is inefficient, fails to unveil intrinsic regulatory mechanisms, and cannot offer scientific guidance or interpretable analytical tools for rational optimization. To establish more precise structure-property relationships and guide composition optimization, researchers have focused on microstructural characterization techniques. Nuclear Magnetic Resonance (NMR) spectroscopy, particularly ^19^F-NMR, is a powerful tool for the precise characterization of fluoroelastomer molecular structures [18,19]. Comprehensive NMR characterization protocols for VDF-TFE-PMVE terpolymers have been established, providing detailed spectral assignments for structural analysis [19]. The macroscopic properties of fluoroelastomers are fundamentally determined by their microscopic molecular structures, which represent a core principle in materials science. ^19^F-NMR, as the most effective technique for characterizing fluorinated compound molecular structures, can comprehensively reflect key structural parameters including monomer feed ratios, sequence distribution, and functional group content. However, extremely complex, multi-scale, nonlinear mapping relationships exist between microscopic NMR structural information and macroscopic property performance.

Current scientific understanding is primarily limited to direct structure–property correlations. For instance, da Cunha et al. [20] confirmed that PMVE’s OCF_3_ groups participate in peroxide vulcanization reactions, while Boyer et al. [21] found that VDF copolymers containing PMVE exhibit glass transition temperatures ranging from −63 to −35 °C. Yuan et al. [9] demonstrated that in poly(VDF-ter-TFE-ter-PMVE) terpolymers, decreased VDF content reduces crystallinity, leading to lower tensile strength but higher elongation at break. However, these known explicit relationships can only explain a small fraction of the observed property variations. Numerous implicit relationships—including multi-body interactions, synergistic effects, and threshold phenomena—remain as cognitive blind spots in the field. Although we cannot fully elucidate all underlying mechanistic pathways, the causal relationships between NMR structural information and macroscopic properties objectively exist. As demonstrated by Twum et al. [22] through their multidimensional ^19^F-NMR techniques for resolving fine sequence structures of poly(VDF-ter-HFP-ter-TFE) terpolymers, NMR spectra contain complete structural fingerprints of materials, which necessarily harbor critical factors that determine macroscopic properties. With recent advances in data science and machine learning technologies, it has become possible to uncover these hidden structure–property correlations through advanced modeling approaches, even in the absence of complete physicochemical mechanistic explanations. Similarly, Duan et al. [23] investigated how different end-group structures affect fluoroelastomer properties through comprehensive end-group analysis. Although these studies have made significant progress in understanding local structure–property relationships, significant limitations persist in composition optimization: current research remains largely qualitative, lacking quantitative models of influence pathways to guide formulation adjustments; the rich structural information contained in NMR spectra remains underutilized, with many unassigned spectral regions potentially holding critical “structural fingerprint” information; and comprehensive modeling frameworks that correlate multidimensional microstructural features with macroscopic properties to provide clear regulatory strategies for composition optimization are absent [24].

In recent years, successes in applying machine learning to materials science [25,26] have offered new avenues for fluoroelastomer composition optimization, with recent studies demonstrating the effectiveness of interpretable ML models for fluoroelastomer property prediction [27]. For example, Sumpter et al. [28] utilized deep neural networks to predict polymer thermal properties, and Kim et al. [29] developed the Polymer Genome platform, advancing materials design through integrated machine learning algorithms. However, the unique complexities of fluoroelastomers present new challenges for existing machine learning methods. Fluoroelastomer synthesis involves harsh conditions (e.g., high temperature and pressure), making sample preparation costly and requiring strict experimental control. Furthermore, fluoroelastomer performance data typically exhibit low variability, making subtle performance changes difficult for traditional regression models and machine learning methods to capture [30]. These challenges, combined with other unique characteristics of fluoroelastomers, pose considerable challenges for conventional modeling approaches in this domain.

While traditional models like linear regression and decision trees offer interpretability, they often lack accuracy with complex nonlinear relationships. Conversely, black-box models (e.g., simple neural networks, ensemble learning) may lack interpretability, a deficiency this study addresses. Much existing research remains at the statistical correlation level, lacking interpretability within the fluoroelastomer composition optimization process. This makes it challenging to elucidate how monomer feed ratios influence final properties via microstructural changes, thereby hindering the provision of interpretable, mechanism-driven analytical tools [31,32]. Iwasaki et al. emphasized that machine learning in materials science should provide “complete explanatory pathways from input to output” rather than merely delivering prediction results [33].

To address these challenges, two-stage modeling strategies have shown significant advantages in chemical process modeling by decomposing complex mapping problems into more manageable sub-problems [34]. For instance, Chen et al. [35] proposed a two-stage machine learning model for alloy corrosion prediction that markedly improved accuracy despite small sample sizes. Kieser et al. [36] explored optimized two-stage designs, showing that rational design and validation boundaries can effectively control error rates and enhance estimation accuracy. These studies offer new perspectives for fluoroelastomer composition optimization analysis, especially for modeling complex data. Meanwhile, range-aware feature engineering offers novel solutions for identifying critical features from low-variability data by assessing the ability of features to discriminate between different ranges of the target variable. Khasidashvili et al. [37] introduced a feature range analysis method that improved prediction accuracy and critical feature identification in low-variability data by generating “range features.” Additionally, Oyamada [38] proposed the APA-tree method, which accelerated range aggregation queries in feature engineering, reducing I/O operations and enhancing data analysis efficiency.

**Figure 1:**
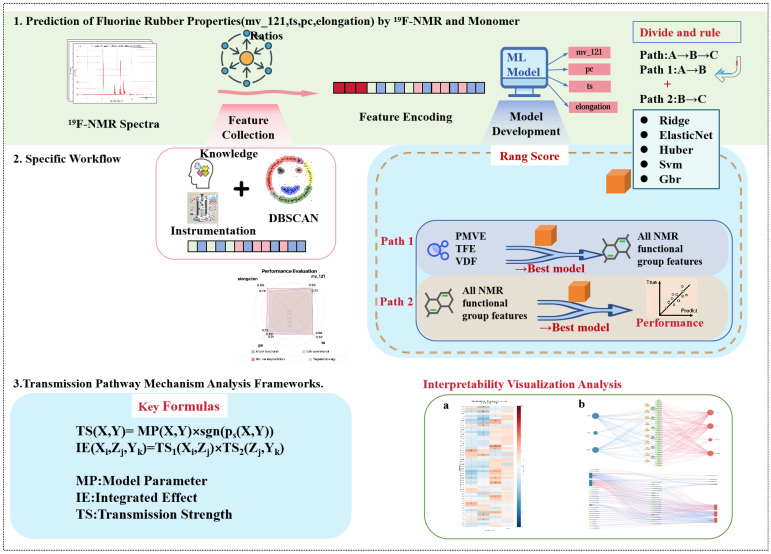
Range-Aware Two-Stage (RATS) modeling framework for fluoroelastomer structure–property analysis. (**a**) Heatmap of correlation analysis; (**b**) Network diagram of transmission pathways. The framework integrates a divide-and-conquer strategy, feature engineering workflow, and transmission pathway analysis.

This study introduces the Range-Aware Two-Stage (RATS) modeling approach, specifically designed to address the dual challenges of fluoroelastomer optimization: extracting predictive patterns from low-variability industrial data while maintaining mechanistic interpretability through NMR structural intermediates. The approach provides three methodological contributions: (1) range scoring technique that identifies predictive features overlooked by traditional correlation analysis (demonstrated through the F7_area case: Pearson correlation 0.01, yet the highest contribution 0.18 to elongation prediction); (2) physically meaningful two-stage decomposition leveraging NMR structural characterization to bridge composition and properties; and (3) systematic pathway quantification enabling mechanism-guided optimization. We identified 72 statistical transmission pathways that inform composition optimization. The approach is grounded in the fundamental materials science principle that macroscopic properties of fluoroelastomers are determined by their microscopic molecular structures. ^19^F-NMR spectroscopy provides comprehensive structural characterization, enabling systematic analysis of composition–structure–property relationships through quantifiable structural intermediates rather than direct empirical correlations. Figure 1 illustrates the overall RATS framework.

## 2. Materials and Methods

Figure 1 illustrates the comprehensive framework of the Range-Aware Two-Stage (RATS) modeling approach for fluoroelastomer structure–property prediction developed in this study. The methodology encompasses four hierarchical components: (1) The top section demonstrates the divide-and-conquer strategy for predicting fluoroelastomer properties using ^19^F-NMR spectral data and monomer feed ratios, decomposing the complex cross-scale mapping into two physically meaningful sub-processes. (2) The middle section presents the systematic feature engineering workflow, integrating domain knowledge with DBSCAN (Density-Based Spatial Clustering of Applications with Noise) clustering to construct a comprehensive microstructural characterization system. (3) The bottom section establishes multidimensional interpretable analysis pathways, including single-stage correlation analysis and dual-stage statistical interpretation networks. (4) The framework enables the transition from “black box prediction” to “white box interpretation” through transparent influence pathway visualization, achieving full-chain mechanistic analysis from monomer feed ratios to macroscopic properties.

### 2.1. Dataset and Preprocessing

#### 2.1.1. Data Collection

This study utilized 52 industrial-scale datasets from poly(VDF-ter-TFE-ter-PMVE) terpolymer fluoroelastomer production, encompassing complete formulation, microstructural, and performance data. The approach is based on the fundamental materials science principle that macroscopic properties are determined by microscopic molecular structures, and ^19^F-NMR spectroscopy provides comprehensive structural characterization of fluoroelastomers. Each dataset included monomer feed ratios (initial molar percentages of PMVE, VDF, and TFE monomers in mol%, constrained by Σmonomer ratio = 100%), four key performance indicators—Mooney viscosity ML(1+10) at 121 °C (mv_121), tensile strength (ts), compression set (pc), and elongation at break (elongation)——where the terms in parentheses are abbreviations, and high-resolution ^19^F-NMR spectral data were acquired using a Bruker Avance Neo 400 MHz nuclear magnetic resonance spectrometer (Bruker Corporation, Billerica, MA, USA) with TopSpin 4.0 software(400 MHz, chemical shift range −218.56 to +18.56 ppm, resolution 0.01 ppm). Analysis focused on regions exhibiting strong resonance signals suitable for quantitative modeling. All samples were prepared and tested following industrial standardized procedures (specific operational parameters and monomer contents are withheld due to confidentiality agreements). Note: This study uses feed ratios as input variables to establish predictive influence pathways rather than determining actual polymer compositions.

**Figure 2:**
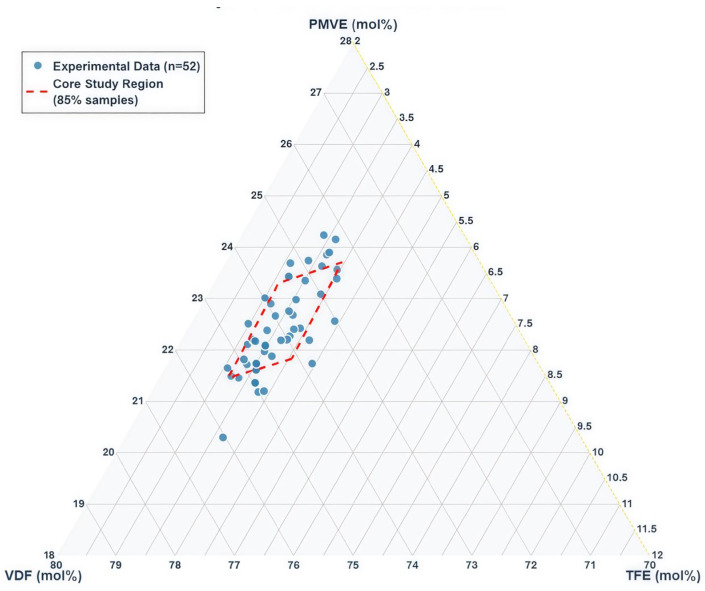
Ternary composition distribution showing monomer feed ratios (mol%, n = 52). Red boundary: core study region (85% samples). Orange line: elastomeric reference. All compositions represent industrially validated formulations.

Figure 2 shows the ternary composition distribution of all 52 samples. The complete dataset spans VDF: 72.22–76.04 mol%, TFE: 2.98–4.44 mol%, PMVE: 19.52–24.80 mol% (all compositions normalized to sum to 100 mol%). These composition ranges represent industrially validated elastomeric formulations. As discussed by Schmiegel [19], VF2 copolymers with perfluorinated comonomers (HFP, TFE, PMVE) require sufficient VDF content to provide the polymer backbone, while the perfluorinated comonomers serve to disrupt VDF crystallinity and enable crosslinking. Our data cluster within established elastomeric composition windows, with 85% of samples concentrated in a narrow range (VDF: 73.7–75.2 mol%, TFE: 3.2–3.8 mol%, PMVE: 21.0–23.1 mol%), validating the necessity of RATS methodology for low-variability data modeling. The composition space excludes the PMVE-TFE axis (VDF = 0%) as VDF forms the essential polymer backbone for elastomeric properties.

#### 2.1.2. Data Preprocessing

All the samples were prepared following industrial standardized procedures. The NMR quantification methodology follows established protocols recommended by fluoroelastomer characterization experts, providing reliable monomer composition determination with validated accuracy. NMR data underwent a standard preprocessing workflow for quality control, including Savitzky–Golay filtering (noise reduction), baseline correction [39], and intensity normalization (details in Supplementary Material S1.1).

### 2.2. Range-Aware Feature Engineering

The complexity of structure–property relationships in fluoroelastomers is manifested in the rich but challenging NMR spectral data. We extracted 66 structural features from ^19^F-NMR spectra. These comprised 42 known functional groups (9 area features and 33 intensity features, Table 1), 15 unknown features (U1-U15, Table 2), and 9 range-aware features (Section 2.2.2). While some relationships are explicit (e.g., PMVE’s OCF_3_ groups affecting cure kinetics [9]), many remain implicit, requiring sophisticated feature engineering to extract meaningful patterns.

#### 2.2.1. Range Score (RS) Metric

Traditional correlation-based feature selection methods often fail to identify critical features in low-variability fluoroelastomer data, where subtle structural differences can significantly impact performance despite weak linear correlations. This challenge reflects the complex, nonlinear nature of structure–property relationships, where important influence pathways may be hidden within seemingly insignificant spectral features.

We proposed the range score (RS) to quantify a feature’s ability to discriminate target variable ranges. It is calculated as the ratio of the global standard deviation of the target variable to the average standard deviation within quartile-based groups of the feature, as shown in Equation (1):

(1) $$RS(X,y)=\frac{\sigma_{{\mathrm{global}}^{2}}(y)}{\frac{1}{k}\sum_{i=1}^{k}\;\;\sigma_{{\mathrm{group}}_{i}}(y)}$$

where $\sigma_{\mathrm{global}}(y)$ represents the global standard deviation of the target variable, $\sigma_{\mathrm{group}}(y)$ denotes the standard deviation of the target variable y within the i-th quartile group of feature X, and k is the number of quartile groups. A higher RS indicates stronger discriminative power for the target variable. This mechanism was inspired by the “purity gain” concept from random forest variable importance assessment by Strobl et al. [40].

A composite score (CS) was developed using Equation (2), combining RS (70% weight) with absolute Pearson correlation (30% weight) to balance statistical correlation with range discrimination ability:

(2) CS(X,y)=0.7×RS(X,y)+0.3×|Corr(X,y)|

This weighting strategy, informed by research from Yu and Liu [41] on feature selection and correlation redundancy, emphasizes range discrimination while considering statistical correlation. The 0.7 and 0.3 weights were determined empirically through validation experiments involving multiple scoring group configurations.

#### 2.2.2. Feature System Construction

Based on ^19^F-NMR spectra, three complementary feature categories were established:

Known functional group features: Based on ^19^F-NMR experimental characterization and literature reports [20,21,22], 9 key chemical shift regions (e.g., PMVE-OCF_3_ groups: −52.9 to −55.3 ppm; CF_2_ backbone segments spanning multiple regions: −126.91 to −128.05 ppm and −92 ppm, encompassing various chain environments) and 33 specific peak intensities were identified (Table 1).

Monomer molar percentages are calculated from integrated NMR peak areas using the following Equation (3):

(3) $$\begin{array}{rr} VDF(\;mol\%) & =\left(\frac{\sum A_{VDF}}{A_{total}}\right)\times 100\%\\ TFE(\;mol\%) & =\left(\frac{\sum A_{TFE}}{A_{total}}\right)\times 100\%\;\\ \;\;PMVE(\;mol\%) & =\left(\frac{\sum A_{PMVE}}{A_{total}}\right)\times 100\% \end{array}$$

where ∑A_VDF represents the sum of all VDF-characteristic peak areas, ∑A_TFE represents all TFE-characteristic peak areas, ∑A_PMVE represents all PMVE-characteristic peak areas, and A_total = ∑A_VDF + ∑A_TFE + ∑A_PMVE. The specific peak area assignments and normalization procedures follow established protocols for fluoroelastomer NMR quantification.

Unknown structural fingerprints: Dynamic DBSCAN clustering identified high-signal regions not covered by known features (0.2 ppm precision). After excluding regions with peak area ratios <0.1% of the total spectrum or <5 data points, 15 unknown interval features (U1–U15) were extracted as unassigned “structural fingerprints”. To provide a chemical context for these uncharacterized regions, systematic chemical shift proximity analysis was conducted by comparing with established assignments from Table 1, with tentative structural hypotheses presented in Table 2 (details in Supplementary Material S1.2).

#### 2.2.3. Construction of Encoded Feature Matrix

Selected known region peak areas (F1, F2,…), known point feature signal intensities (F1*, F2*,…), unknown region peak areas (U1, U2,…), and range-aware features (R1, R2,…) were combined to construct a unified feature matrix X, as shown in Equation (4):

(4) $$\begin{array}{r} X=\left[\begin{array}{ccccccccccccc} r & F & F & U & R & & & & & & & & \\ r & F_{1} & \ldots & F_{6} & F_{1*} & \ldots & F_{32*} & U_{1} & \ldots & U_{15} & R_{1} & \ldots & R_{9}\\ \vdots & \vdots & \ddots & \vdots & \vdots & \ddots & \vdots & \vdots & \ddots & \vdots & \vdots & \ddots & \vdots \\ r & F_{n} & \ldots & F_{6+n} & F_{n*} & \ldots & F_{32+n*} & U_{n} & \ldots & U_{15+n} & R_{n} & \ldots & R_{9+n} \end{array}\right] \end{array}$$

where r includes r_1_, r_2_, r_3_ for 3 monomer feed ratios, with 9 known region peak area features, 33 known point feature intensity features, 15 unknown features, and 9 range-aware features.

Prior to model training, all numerical features were normalized using StandardScaler Equation (5), transforming them to a standard normal distribution (mean 0, standard deviation 1). This normalization mitigated the “dimensional effect” in polymer feature data, as noted by Chen and Guestrin [42], preventing features with larger magnitudes from disproportionately influencing model training.

(5) $$\overset{ˆ}{x}=\frac{x-\mu }{\sigma }$$

where μ is the feature mean and σ is the standard deviation.

### 2.3. Two-Stage Modeling Implementation

#### 2.3.1. Modeling Foundation

Algorithm Integration: To maintain model consistency and facilitate comparability, both stages utilized the same core modeling components, thereby reducing overall complexity. We integrated five algorithms renowned for strong performance in materials science and other fields: Ridge Regression, ElasticNet Regression, Huber Regression, Support Vector Regression (SVR), and Gradient Boosting Regression (GBR). Grid search was used for hyperparameter optimization to enhance model performance and interpretability.

Range Scoring (RS) Technique: As previously defined (Equation (1)), this technique groups feature values by quartiles and calculates the ratio of the global standard deviation to the average within-group standard deviation. The composite score (Equation (2)) combines RS (70%) and statistical correlation (30%), balancing feature discrimination with linear association. This effectively addresses modeling challenges from low-variability fluoroelastomer data. In stage one, RS assesses monomer influence on structural features; in stage two, it screens structural features most predictive of performance.

#### 2.3.2. First Stage Modeling: Feed Ratio → NMR Feature Mapping

The first stage established mappings from the 3 monomer feed ratios to each of the 66 NMR structural features. This stage employed a unified input strategy (monomer feed ratios as input), building independent prediction models for each NMR feature. The focus was on establishing stable and reliable fundamental ratio-structure mappings, without applying target transformation, extreme value resampling, or other data augmentation techniques.

#### 2.3.3. Second Stage Modeling: NMR Feature → Property Mapping

The second stage constructed property prediction models using the 66 NMR features (predicted from the first stage) as input, to predict four key performance indicators (mv_121, ts, pc, elongation). This stage incorporated target transformation, extreme value resampling, and dimensionality reduction techniques.

Four distinct modeling strategies were employed: (1) a domain knowledge-based strategy selecting functional group features based on chemical rules; (2) a statistical significance strategy using *p*-value screening for relevant features; (3) a multivariate prediction strategy using Partial Least Squares (PLS) regression to manage feature redundancy; and (4) a targeted strategy customizing feature combinations based on specific performance characteristics. Each strategy was trained independently, and cross-validation selected the optimal configuration as the best prediction model for each property.

The two stages were linked by a rigorous data transfer workflow: the 66 NMR feature predictions from stage one served directly as input for stage two, thus constructing a complete influence pathway from monomer feed ratios, through microstructures, to macroscopic properties.

#### 2.3.4. Training and Evaluation Strategy

Both stages employed Leave-One-Out Cross-Validation (LOOCV) for robust evaluation. In each of the 52 iterations, 51 samples were used for training and the remaining sample for testing, yielding unbiased performance estimates.

Evaluation metrics consistently included the coefficient of determination (R^2^), Root Mean Square Error (RMSE), and RMSE percentage. Effectiveness was further validated using a three-pronged approach: range sensitivity (quantile validation), interpretability (influence pathway analysis), and model performance (R^2^-based selection of optimal configurations).

#### 2.3.5. Grouped Comparison Experiments

To validate the effectiveness of the two-stage modeling approach, two groups of comparative experiments were designed. The first group served as benchmark comparisons, including B1 (direct modeling: monomer feed ratios directly predicting properties without RS indicators), B2 (traditional two-stage modeling without RS indicators), and RATS (addressing low-variability characteristics of fluoroelastomer performance data). The second group involved quantile stratified validation, dividing performance data into low (<Q1), medium (Q1–Q3), and high (>Q3) quantile intervals. Prediction accuracy was compared across different performance intervals, with emphasis on evaluating the improvement effects of range-aware techniques in extreme value regions.

### 2.4. Transmission Pathway Analysis Method

This study established an influence pathway identification method based on the dual-stage machine learning framework to quantify the complete action mechanisms from monomer composition to performance metrics. The approach systematically mapped the complex cross-scale relationships through transparent mechanistic interpretation.

The influence strength was defined as the product of model parameters and correlation direction, as expressed in Equation (6):

(6) TS(X,Y)=MP(X,Y)×sgn(ρs(X,Y))

To ensure robust influence strength quantification across different optimal model types identified through cross-validation, we developed an adaptive parameter extraction strategy that maintains consistency in pathway interpretation. Model parameter extraction (MP) depended on the optimal model type, as specified in Equation (7):

(7) $$\begin{array}{ll} & MP(X,Y)=\{\\ & FI(X,Y),\;\;\;\;\;\;\;\;\;for\;tree-based\;models\\ & |\beta (X,Y)|,\;\;\;\;\;\;\;for\;linear\;models\\ & 0,\;\;\;\;\;\;\;\;\;\;\;\;\;\;\;\;\;\;\;\;\;otherwise\\ & \} \end{array}$$

where the model parameter extraction adapts to different algorithm types to ensure consistent interpretation across diverse modeling approaches. FI(X, Y) represented the feature importance score of feature X for target Y in tree-based ensemble models (gradient boosting, random forest), calculated through information gain to quantify the contribution of each feature to prediction accuracy; β(X, Y) denoted the regression coefficient of feature X in linear regression models (Ridge, ElasticNet, Huber regression), representing the direct linear relationship strength between features and targets; ρs(X, Y) was the Spearman rank correlation coefficient between X and Y, chosen over Pearson correlation to capture monotonic relationships regardless of linearity, which is particularly important for fluoroelastomer data exhibiting complex nonlinear patterns; sgn(·) represented the sign function defined in Equation (8) to preserve the directional nature of influences:

(8) $$sgn(x)=\left\{\begin{array}{ll} +1, & \mathrm{if}\;x>0\\ 0, & \mathrm{if}\;x=0\\ -1, & \mathrm{if}\;x<0 \end{array}\right.$$

The complete influence pathway construction involved the following systematic steps: (1) mapping features from 52 monomer models to generate TS_1_ in the first-stage NMR prediction; (2) mapping features from 4 NMR models to generate TS_2_ in the second-stage performance prediction; (3) connecting the two stages via common NMR mediator features to form complete pathways; (4) calculating the composite influence coefficient as specified in Equation (9):

(9) $$IE(Xi,Zj,Yk)=TS_{1}(Xi,Zj)\times {TS}_{2}\left({Zj}_{j},Yk\right)$$

where IE represents the composite influence coefficient, quantifying the overall effect magnitude of a complete monomer → structure → property pathway

Significance thresholds were applied to filter effective influence pathways, as defined in Equation (10):

(10) $$\Psi \left(X_{i},Z_{j},Y_{k}\right)=I\left(\left|TS_{1}\left(X_{i},Z_{j}\right)\right|>\tau_{1}\right)\wedge I\left(\left|TS_{2}\left(Z_{j},Y_{k}\right)\right|>\tau_{2}\right)$$

where I(·) represented the indicator function, and τ_1_ = τ_2_ = 0.05 were established as significance thresholds. Based on the sign of the composite influence coefficient, pathways were classified according to Equation (11):

(11) $$Type\left(X_{i},Z_{j},Y_{k}\right)=\left\{\begin{array}{ll} \mathrm{Promoting}, & \mathrm{if}\;\mathrm{IE}\left(X_{i},Z_{j},Y_{k}\right)>0\\ \mathrm{Inhibiting}, & \mathrm{if}\;\mathrm{IE}\left(X_{i},Z_{j},Y_{k}\right)<0 \end{array}\right.$$

This methodology enabled the systematic identification and quantification of bidirectional regulatory mechanisms, providing transparent mechanistic insights into fluoroelastomer composition optimization through comprehensive pathway analysis.

### 2.5. Research Scope and Limitations

This study focuses on establishing mechanistic influence pathways between monomer feed ratios and final properties through NMR-derived structural features. The approach establishes quantitative pathways through model parameter analysis rather than simple correlation. However, pathways involving unknown structural features (Chemical shift assignments focus on spectral regions with strong, reproducible signals (>5% relative intensity). ^2^ Rf = fluoroalkyl chain segments; * = observed fluorine nucleus. ^3^ Complete sequence determination remains technically challenging for complex systems).

Table 2 relies on chemical shift proximity analysis and requires experimental validation for definitive structural confirmation. The approach aims to provide interpretable regulatory mechanisms for composition optimization rather than quantitative composition analysis. Actual polymer compositions are not determined due to industrial confidentiality constraints.

## 3. Results and Discussion

### 3.1. Two-Stage Modeling Performance Evaluation

#### 3.1.1. First Stage: Ratio → NMR Feature Mapping

The range-aware models for the first stage (feed ratio → NMR features) demonstrated excellent performance. Table 3 presents the prediction performance metrics for representative NMR feature types.

The results indicate that even unknown structural fingerprints were predicted with high precision, demonstrating stable and predictable relationships between monomer feed ratios and microstructural characteristics.

#### 3.1.2. Second Stage: NMR Features → Property Mapping

The RATS model’s property prediction accuracy was compared with a range-aware direct modeling approach (monomer feed ratios directly predicting properties). Results are shown in Table 4.

RATS achieved an average R^2^ of 0.90 across four property predictions, representing a 0.14 improvement over direct modeling and a 28% reduction in prediction error. These improvements were particularly significant in extreme value regions (<Q1 and >Q3 quartiles), where RATS achieved 50% better prediction accuracy compared to traditional methods—crucial for material optimization where extreme values often define application boundaries and breakthrough opportunities.

### 3.2. Model Overall Evaluation Methods

#### 3.2.1. Baseline Comparison Evaluation

Table 5 compares the performance of RATS with baseline methods. RATS significantly outperformed both direct modeling (B1) and traditional two-stage modeling (B2) across all indicators. RATS achieved an average R^2^ of 0.90, an improvement of approximately 0.11 over B1 and 0.13 over B2. These results demonstrate RATS’s superior capability in handling low-variability data challenges.

#### 3.2.2. Quantile Stratified Performance Analysis

**Figure 3:**
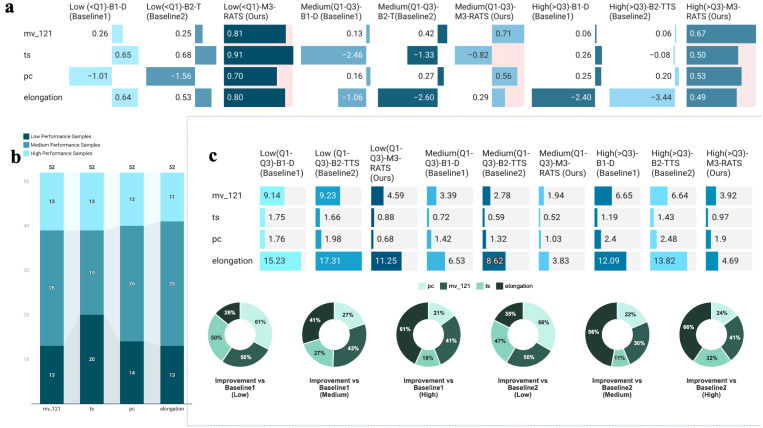
(**a**) Prediction accuracy (R^2^) of the three methods across performance quantile regions. (**b**) Sample distribution across performance quantile regions. (**c**) Top: Relative error of the three methods across quantile regions. Bottom: Improvement of RATS compared to B1 and B2.

Figure 3 demonstrates the prediction accuracy of three methods across different performance quantile intervals. RATS performed particularly outstandingly in extreme intervals. In Figure 3a, our RATS method’s error bars in extreme intervals (<Q1 first 25%, >Q3 latter 25%), such as mv_121’s (0.81, 0.67), significantly outperformed B1 (0.26, 0.06) and B2 (0.25, 0.06). Figure 3c shows that RATS achieved 4.59% relative error in the first 25% (<Q1) region (using mv_121 as an example), representing 50% improvement over direct modeling (B1) and 50% improvement over traditional two-stage modeling (B2), with similar results for the latter 25% (>Q3). This enhanced prediction at performance extremes is crucial for material composition optimization, as these extremes often define a material’s application boundaries, such as usable lower limits (first 25%) and breakthrough upper limits (latter 25% >Q3).

#### 3.2.3. RS Effectiveness

**Figure 4:**
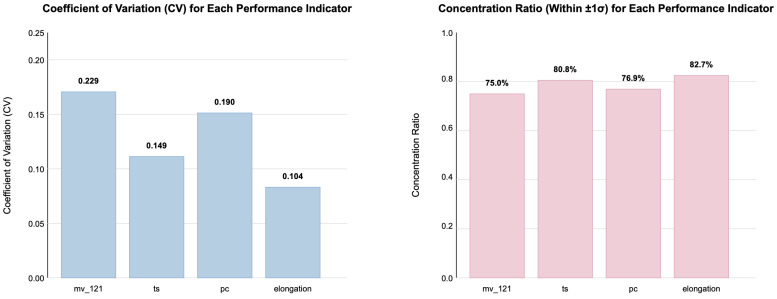
(**Left**) Analysis of the narrow distribution characteristics of fluoroelastomer performance indicators (annotated with CV and concentration ratios). (**Right**) Comparison of the coefficient of variation (CV) and concentrated distribution ratios for these indicators.

Systematic statistical analysis of fluoroelastomer performance data (mv_121, ts, pc, elongation) was conducted. Quantitative coefficient of variation (CV) analysis (Figure 4, left; CV values 0.104–0.229) and concentrated distribution ratios (Figure 4, right; 75.0–82.7% of data within mean ± 1σ) revealed that all performance indicators exhibit significantly low variability. This characteristic often renders traditional feature selection methods ineffective.

Our range-aware method, utilizing RS, successfully identified key features overlooked by methods like Pearson correlation analysis (Table 6).

This demonstrates the range score effectiveness through a compelling example: F7_area, virtually ignored by traditional methods (Pearson correlation: 0.01, ranked 47th), emerged as the most predictive feature (range score rank: 1) with the highest contribution (0.18) to elongation prediction. This dramatic reversal—from statistically insignificant to most important—exemplifies RATS’s capability to identify critical patterns hidden within low-variability data, addressing a fundamental limitation of conventional correlation-based approaches.

### 3.3. Analysis of Conductive Pathway Mechanisms

#### 3.3.1. Conductive Pathway Identification Results

**Figure 5:**
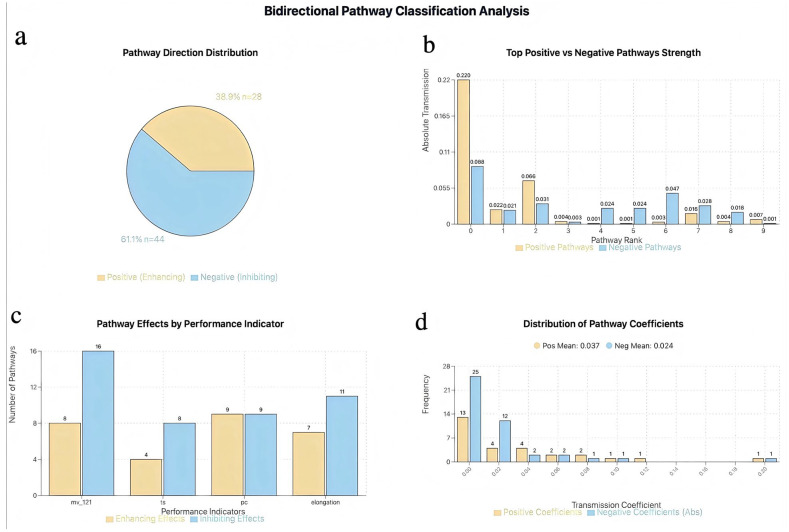
Comprehensive pathway analysis: A total of 72 transmission routes governing fluoroelastomer performance with asymmetric regulatory patterns (61.1% Negative vs. 38.9% Positive). (**a**) Pathway direction distribution, (**b**) comparison of the top 8 strongest influence pathways, (**c**) number of pathways for each performance indicator, and (**d**) influence coefficient distribution.

Based on the methodology established in Section 2.4, influence pathways of the two-stage model were systematically identified. From a theoretical total of 792 pathways (calculated as 3 × 66 × 4 = 792, where 66 represents the total NMR features), 72 complete influence pathways were identified through bidirectional pathway analysis (Figure 5a). The classification results revealed significant mechanistic differences, leading to the formation of 72 fully connected pathways.

The influence characteristics differed significantly among performance indicators (Figure 5c). Mooney viscosity (mv_121) was most strongly affected by negative pathways (16 negative versus 8 positive), elongation showed moderate negative dominance (11 negative versus 7 positive), while compression set (pc) exhibited the most balanced positive and negative pathways (9 positive versus 9 negative), requiring precise ratio control. The influence coefficient distribution revealed that the average effect of positive influences (0.037) slightly exceeded that of negative influences (0.024), yet the numerical advantage of negative pathways enabled them to dominate overall regulation. Figure 5b presents the characteristics of the top 8 strongest positive and negative influence pathways, showing the strongest positive coefficient of 0.220 and the strongest negative absolute value of 0.088. Positive pathways demonstrated higher peak strength but exhibited steep decay from 0.220 to 0.001, while negative pathways showed more moderate but stable strength distribution from 0.088 to 0.003, reflecting the robustness and consistency of the negative influence mechanism across multiple pathways. Figure 5d further validated that while the positive average effect (0.037) slightly exceeded the negative (0.024), negative pathways dominated overall regulation due to their numerical advantage and stable strength distribution.

#### 3.3.2. Key Transmission Pathway Identification and Quantitative Analysis

**Figure 6:**
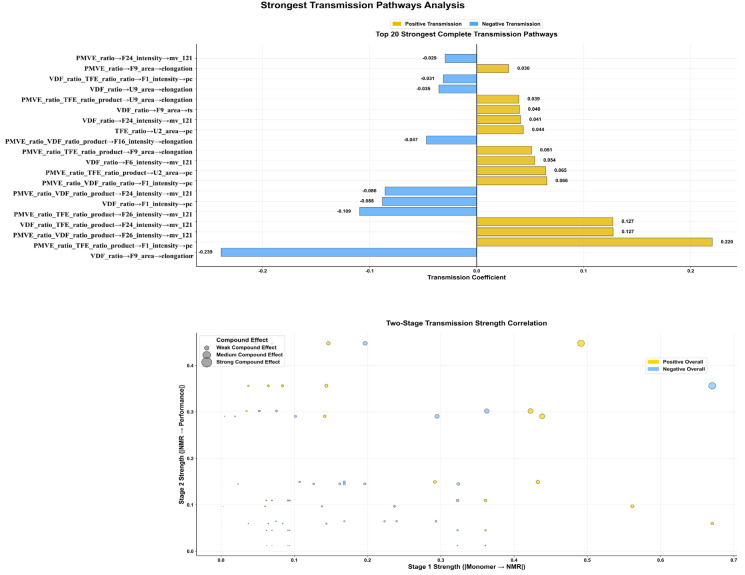
Key influence pathway identification and two-stage strength correlation analysis. (**Top**) Ranking of the top 20 influence pathway strengths. (**Bottom**) Two-stage influence strength correlation validation.

Figure 6 presents the 20 key pathways with the highest influence strengths, revealing specific molecular regulatory mechanism pathways. The strongest positive pathway (PMVE_ratio × TFE_ratio → F1_intensity → compression set, IE = +0.220) demonstrates systematic regulation through well-characterized structural features. F1_intensity (−146.59 ppm) corresponds to -CH_2_-CF_2_-CF*-(OCF_3_)-CF_2_-CH_2_- structures [9,21,43], providing structural validation for the observed statistical relationship. This pathway strength quantifies the synergistic effect between PMVE and TFE ratios on compression set performance, offering precise guidance for composition adjustment: increasing PMVE and TFE interaction enhances compression set resistance through specific structural modifications. The observed pathway aligns with established chemical knowledge, as da Cunha et al. [20] confirmed that PMVE’s OCF_3_ groups participate in peroxide vulcanization reactions, and the quantified influence strength (IE = +0.220) represents the transmission effect through the two-stage modeling framework rather than simple correlation. The strongest negative influence pathway was quantified as VDF_ratio → F9_area → elongation (IE = −0.219). This pathway indicates that VDF content influences elongation at break through F9_area signals, consistent with Yuan et al.’s [9] findings that decreased VDF content affects crystallinity and mechanical properties. The negative influence strength reflects the systematic transmission effect within the modeling framework. A significant influence pathway involving unassigned spectral features was identified: TFE_ratio → U2_area → pc (IE = +0.044). Chemical shift proximity analysis (Chemical shift assignments focus on spectral regions with strong, reproducible signals (>5% relative intensity). ^2^ Rf = fluoroalkyl chain segments; * = observed fluorine nucleus. ^3^ Complete sequence determination remains technically challenging for complex systems.

Table 2 suggests U2_area (−115.80 ppm) may correspond to-(CH_2_-CF_2_)-(CF_2_-CH_2_)- related structures based on minimal deviation (Δδ = −0.1 ppm) from F14_intensity (−115.7 ppm). While this tentative assignment provides a structural hypothesis for the observed pathway, definitive confirmation requires 2D NMR spectroscopy and additional validation studies. The quantified pathway strength indicates a systematic optimization route for TFE content effects on compression set performance, offering practical guidance for composition adjustment while acknowledging the need for structural validation.

The two-stage influence strength correlation analysis (Figure 6, bottom) showed that strong influence pathways maintained high consistency across both monomer→NMR and NMR → performance stages, validating the reliability of the two-stage modeling approach. Pathways with stronger composite effects (large dots) were primarily concentrated in the positive influence region, indicating higher efficiency of positive pathways.

#### 3.3.3. Complete Transmission Network Mechanism Analysis

**Figure 7:**
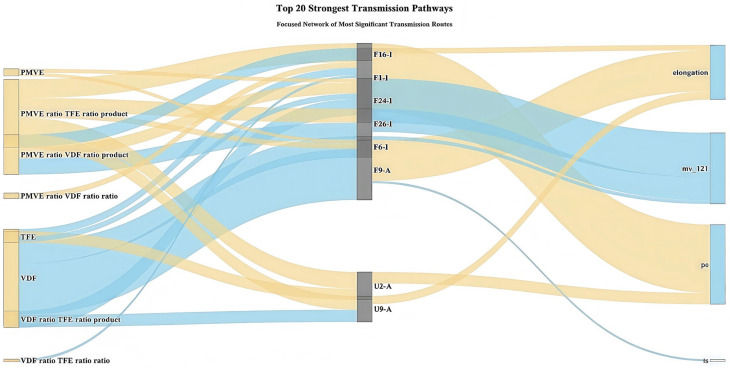
Sankey diagram of core influence mechanisms for fluoroelastomer formulation regulation, where yellow represents positive overall influence pathways and blue represents negative overall influence pathways.

Based on Sankey diagram analysis (Figure 7), a systematic mechanistic understanding of fluoroelastomer composition regulation (the systematic control of monomer feed ratios to achieve desired properties) was provided, revealing the core influence mechanisms. PMVE-related ratios dominated positive influence flows (yellow), primarily regulating compression set and Mooney viscosity through OCF_3_-related signals such as F1-I (F1_intensity) and F26-I (F26_intensity). VDF ratios dominated negative influence flows (blue), significantly affecting elongation at break primarily through F9-A (F9_area) flexible segment signals. This focused network revealed the most critical “composition–structure–property” influence pathways, providing precise regulatory targets for composition optimization.

#### 3.3.4. Mediation Effect Validation of Two-Stage Modeling

**Figure 8:**
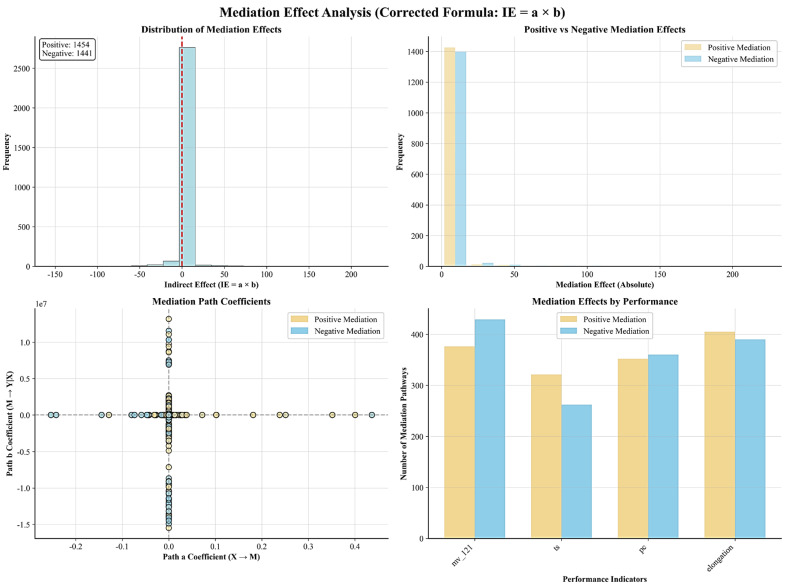
Statistical validation analysis of mediation effects in two-stage modeling, showing distribution of positive and negative mediation effect pathways and comparison of mediation dependency degrees for each performance indicator.Dashed lines indicate: the vertical red line at x = 0 separates positive and negative mediation effects, while the horizontal/vertical gray lines at x = 0 and y = 0 mark the origin of path coefficients (a and b).

To validate the effectiveness of the two-stage modeling approach, statistical verification of influence pathways was conducted using the corrected mediation effect formula (IE = a × b) (Figure 8). The analysis revealed 1454 positive mediation effect pathways and 1441 negative mediation effect pathways, showing nearly balanced distribution and validating both the bidirectionality of influence mechanisms and statistical reliability.

Significant differences in mediation intensity among performance indicators were observed. Mooney viscosity and elongation at break exhibited the highest number of mediation pathways (>400 pathways), while tensile strength showed the lowest mediation dependency (approximately 260 pathways), indicating significant differences in the degree of dependence on indirect regulatory mechanisms among different performance indicators.

## 4. Conclusions

This study developed the RATS method for fluoroelastomer composition optimization. The method addresses low-variability data challenges in fluoroelastomer research. The range-aware feature engineering technique effectively addressed the limitations of traditional correlation analysis in feature identification for low-variability data. For instance, while the F7_area feature exhibited only a 0.01 Pearson correlation coefficient, it ranked first in range scoring and contributed most significantly to elongation prediction (0.18), validating the technique’s advantages in uncovering hidden patterns.

The systematic identification of 72 mechanistic pathways through model parameter integration reveals fundamental optimization principles: with 61.1% exhibiting negative regulation versus 38.9% positive effects, fluoroelastomer optimization primarily involves constraint management rather than simple enhancement. The RATS methodology uniquely connects industrial control parameters (feed ratios) to performance outcomes through quantifiable structural intermediates, enabling systematic formulation adjustment without analytical complexity. Key pathways with strong literature validation demonstrate the approach’s reliability: PMVE-related pathways through well-characterized OCF_3_ functional groups (influence strength +0.220) align with established crosslinking chemistry [9,20,21] while VDF-related pathways through backbone flexibility mechanisms (influence strength −0.219), consistent with Yuan et al. [9] findings that decreased VDF content affects crystallinity and elongation at break in poly(VDF-ter-TFE-ter-PMVE) terpolymers. Even pathways involving unassigned spectral features (Chemical shift assignments focus on spectral regions with strong, reproducible signals (>5% relative intensity). ^2^ Rf = fluoroalkyl chain segments; * = observed fluorine nucleus. ^3^ Complete sequence determination remains technically challenging for complex systems).

Table 2 provides statistical optimization guidance, though mechanistic confirmation requires structural validation through 2D NMR spectroscopy or model compound synthesis. This pathway quantification methodology provides systematic guidance for fluoroelastomer optimization decision-making.

The methodological limitations include the relatively small industrial dataset (52 samples), the need for experimental validation of 26% of features lacking definitive structural assignments, and the requirement for broader validation across extended composition ranges. Industrial data confidentiality constraints prevent comprehensive mechanistic validation and limit generalizability assessment. Future research priorities include systematic 2D NMR characterization of unknown features, controlled synthesis validation of proposed pathways, extension to diverse fluoroelastomer systems, and integration of quantum chemical calculations for theoretical structural assignment support.

## Figures and Tables

**Table 1:** ^19^F-NMR feature extraction methods and structural assignments for known functional groups. Chemical shift assignments follow established methodology for quantitative monomer determination in fluoroelastomer terpolymers.

Chemical Shift Range/Point (ppm)	Corresponding Segment Structure	Feature Type	Calculation
−50.30~−52.10	-C***F***(C***F_3_***O)C***F_2_***-	F1_area	Trapezoidal integration
−52.90~−55.30	-C***F***(C***F_3_***O)C***F_2_***-	F2_area	Trapezoidal integration
−57.60~−58.90	-C***F***(C***F_3_***O)C***F_2_***-	F3_area	Trapezoidal integration
−70.80~−78.10	-C***F***(C***F_3_***)C***F_2_***-	F4_area	Trapezoidal integration
92.50~94.40	-CH_2_(C***F_2_***)CH_2_-	F5_area	Trapezoidal integration
108.50~115.10	-C***F_2_***(C***F_2_***)CH_2_-	F6_area	Trapezoidal integration
116.00~120.70	-C***F***-C***F_2_***(C***F_2_***)C***F_2_***-	F7_area	Trapezoidal integration
121.10~125.60	-O-C***F***-C***F_2_***(C***F_2_***)C***F_2_***-	F8_area	Trapezoidal integration
125.70~128.40	-C***F_2_***(C***F_2_***)C***F_2_***-	F9_area	Trapezoidal integration
−146.59	-CH_2_-C***F_2_***-CF*-(OC***F_3_***)-C***F_2_***-CH_2_-	F1-intensity	Peak height
−145.95	-C***F_2_***-C***F2***-CF*-(OC***F_3_***)-CH_2_-C***F_2_***-	F2-intensity	Peak height
−128.05	-Rf-C***F_2_***-C***F_2_***-CF_2_*-C***F_2_***-Rf-	F4-intensity	Peak height
−126.91	-C***F_2_***-C***F_2_***-C***F_2_***-CF_2_*-C***F_2_***-Rf-	F5-intensity	Peak height
−126.80	-[C***F_2_***-C***F***(OC***F_3_***)]-CH_2_-C***F_2_***-	F6-intensity	Peak height
−126.32	-CH_2_-C***F_2_***-C***F_2_***-CF_2_*-C***F_2_***-C***F_2_***-	F7-intensity	Peak height
−124.13	-C***F_2_***-C***F_2_***-C***F_2_***-CF_2_*-C***F_2_***-CH_2_-C***F_2_***-	F8-intensity	Peak height
−123.91	-C***F_2_***-C***F_2_***-C***F***(OC***F_3_***)-CF_2_*-C***F_2_***-	F9-intensity	Peak height
−123.63	-C***F_2_***-C***F***(OC***F_3_***)-CF_2_ *-C***F_2_***-	F10-intensity	Peak height
−123.40	-CH2-C***F2***-CF2*-C***F2***-C***F***(OC***F_3_***)-	F11-intensity	Peak height
−122.50	-[C***F_2_***-C***F***(OC***F_3_***)]-	F12-intensity	Peak height
−116.97	-CH2-CF2*-C***F***(OC***F_3_***)-C***F2***-	F13-intensity	Peak height
−115.70	-(CH_2_-C***F_2_***)-(C***F_2_***-CH_2_)-(CH_2_-C***F_2_***)-	F14-intensity	Peak height
−114.80	-CH_2_-C***F_2_***H-	F15-intensity	Peak height
−114.67	-C***F_2_***-CH_2_-CF2*-C***F_2_***-Rf-	F16-intensity	Peak height
−113.72	-(CH_2_-C***F_2_***)-(C***F_2_***-CH_2_)-(CH_2_-C***F_2_***)-	F17-intensity	Peak height
−113.48	-C***F_2_***-CH_2_-CF_2_*-C***F_2_***-CH_2_-	F18-intensity	Peak height
−112.38	-Rf-CH_2_-CF_2_*-C***F_2_***-CH_2_-	F19-intensity	Peak height
−111.00	-(CH_2_-C***F_2_***)-[C***F_2_***-CF(OC***F_3_***)]-	F20-intensity	Peak height
−110.75	-CH_2_-C***F_2_***-CH_2_-CF_2_*-Rf-	F21-intensity	Peak height
−110.16	-Rf-CH_2_-CF_2_*-C***F_2_***-Rf-	F22-intensity	Peak height
−109.00	-CH_2_C***F_2_***-C***F_2_***CH_2_I-	F23-intensity	Peak height
−95.36	-Rf-C***F_2_***-CH_2_-CF_2_ *-CH_2_-Rf-	F24-intensity	Peak height
−94.80	-(CH_2_-C***F_2_***)-(C***F_2_***-CH_2_)-(CH_2_-C***F_2_***)-(CH_2_-C***F_2_***)-	F25-intensity	Peak height
−94.21	-C***F_2_***-CH_2_-CF_2_ *-CH_2_-Rf-	F26-intensity	Peak height
−92.59	-C***F2***-CH2-CF_2_ *-CH_2_-C***F_2_***-	F27-intensity	Peak height
−92.00	-C***F_2_***-CH_2_-C***F_2_***-CH_2_-C***F_2_***-	F28-intensity	Peak height
−73.00	-C***F_2_***C***F***(OC***F_3_***)I-	F29-intensity	Peak height
−59.00	-C***F***(OC***F_3_***)C***F_2_***I-	F30-intensity	Peak height
−53.17	-C***F_2_***-CH_2_-C***F***(OCF_3_ *)-C***F_2_***-C***F_2_***-	F31-intensity	Peak height
−52.00	-OC***F_3_***	F32-intensity	Peak height
−40.00	-CH_2_C***F_2_***I-	F33-intensity	Peak height

^1^ Chemical shift assignments focus on spectral regions with strong, reproducible signals (>5% relative intensity). ^2^ Rf = fluoroalkyl chain segments; * = observed fluorine nucleus. ^3^ Complete sequence determination remains technically challenging for complex systems.

**Table 2:** Unknown structural features: Chemical shift analysis and tentative assignments (assignment confidence: high (Δδ < 0.5 ppm), medium (Δδ 0.5–1.5 ppm), low (Δδ > 1.5 ppm). Note: Tentative assignments based on chemical shift proximity analysis with known features from Table 1. All structural hypotheses require experimental validation through 2D NMR spectroscopy or model compound synthesis for definitive confirmation.

Unknown Feature	Chemical Shift (ppm)	Closest Known Feature (ppm)	Δδ (ppm)	Tentative Assignment (Assignment Confidence)
U1_area	−125.70	F9_area (−127.2)	+1.50	C***F_2_***-C***F_2_*** variants (Medium)
U2_area	−115.80	F14_intensity (−115.7)	−0.10	-(CH_2_-C***F_2_***)-(C***F_2_***-CH_2_)- variants (High)
U3_area	−115.60	F14_intensity (−115.7)	−0.10	Modified -(CH_2_-C***F_2_***)-(C***F_2_***-CH_2_)- (High)
U4_area	−115.40	F14_intensity (−115.7)	+0.30	-(CH_2_-C***F_2_***)-(C***F_2_***-CH_2_)- environments (High)
U5_area	−115.20	F14_intensity (−115.7)	+0.50	Similar to F14, different context (Medium)
U6_area	−95.52	F24_intensity (−95.36)	−0.16	Related to -C***F_2_***-CH_2_-C***F_2_***- (High)
U7_area	−95.32	F24_intensity (−95.36)	+0.04	Similar CH_2_-C***F_2_*** environments (High)
U8_area	−95.12	F25_intensity (−94.8)	−0.32	Modified -(CH_2_-C***F_2_***)- chains (High)
U9_area	−94.92	F25_intensity (−94.8)	−0.12	C***F_2_***-CH_2_ chain variants (High)
U10_area	−94.72	F26_intensity (−94.21)	−0.51	Related to -C***F_2_***-CH_2_-C***F_2_***- (Medium)
U11_area	−94.52	F26_intensity (−94.21)	−0.31	Similar chain structures (High)
U12_area	−52.90	F32_intensity (−52)	−0.90	OC***F_3_ ***variants (High)
U13_area	−52.70	F32_intensity (−52)	−0.70	Modified PMVE-OC***F_3_*** (High)
U14_area	−52.50	F32_intensity (−52)	−0.50	OC***F_3_*** in different environments (High)
U15_area	−52.30	F32_intensity (−52)	−0.30	PMVE-related OC***F_3_ ***structures (High)

**Table 3:** Performance metrics for first-stage models (feed ratio → NMR Features).

Feature Type	Feature Example	Best Model	R^2^	RMSE	RMSE%
KnoArea	F5_area	gbr	0.93	0.11	1.50
KnoIntensity	F14_intensity	gbr	0.96	0.11	3.60
Unk.Area	U5_area	huber	0.93	0.14	2.10
AvgPerformance	-	-	0.94	0.10	2.40

**Table 4:** Range-aware (two-stage modeling vs. direct modeling) performance comparison.

Performance Metric	Two-Stage Best Strategy	RATS R^2^	RATS RMSE	RS-Direct R^2^	RS-Direct RMSE	Improvement
mv_121	TargetedStrategy	0.92	3.88	0.75	5.31	0.17
ts	MultivariatePrediction	0.92	0.80	0.89	1.18	0.03
pc	MultivariatePrediction	0.92	1.36	0.75	5.67	0.17
elongation	MultivariatePrediction	0.86	7.75	0.68	7.16	0.18
AvgPerformance	-	0.90	3.45	0.76	4.83	0.14

**Table 5:** Three baseline model validations: effectiveness comparison results of two-stage modeling and RATS technology.

Performance Metric	B1 R^2^	B1 RMSE	B2 R^2^	B2 RMSE	RATS R^2^	RATS RMSE	RATS vs. B1	RATS vs. B2
mv_121	0.79	6.14	0.80	6.01	0.92	3.88	0.13	0.12
ts	0.77	1.31	0.77	1.31	0.92	0.80	0.14	0.14
pc	0.84	1.78	0.83	1.83	0.91	1.36	0.07	0.08
elongation	0.79	10.58	0.70	12.46	0.86	7.75	0.10	0.16
AvgPerformance	0.80	4.95	0.78	5.40	0.90	3.45	0.11	0.13

**Table 6:** Top contributing features for elongation prediction: Pearson correlation vs. RS ranking.

Feature	Range_Score	Range_Rank	Abs_Pearson_corr	Corr_Rank	Proj-Contribution
F7_area	1.17	1	0.01	47	0.18
U8_area	1.17	2	0.13	15	0.11
F9_area	1.16	3	0.15	11	0.14
F30_intensity	1.16	4	0.02	44	0.10
U15_area	1.15	5	0.02	45	0.08

## Data Availability

The data that has been used is confidential.

## Supplementary Materials

The following supporting information can be downloaded at: https://www.mdpi.com/article/10.3390/ma18194618/s1, Table S1-1: Savitzky-Golay Filter Parameters; Table S1-2: Baseline Correction (AsLS) Parameters; Table S1-3: Chemical Shift Calibration Standards; Table S1-4: DBSCAN Clustering Parameters; Table S1.2-1: Known Chemical Shift Ranges and Functional Group Characteristics; Table S1.2-2: Known Chemical Shift Characteristic Points and Assignments;Table S1.4-1: Range Score Calculation Framework; Table S1.4-2: Key Functional Group Ratio Features; Table S1.4-3: Range Indicator Construction Strategy; Table S1.4-4: Extreme Sample Identification and Resampling; Table S1.4-5: Feature Integration Hierarchy; Table S1.4-6: Composite Scoring System; Table S1.4-7: Feature Quality Assurance Criteria; Table S1.4-8: Implementation Parameters; Table S1.4-9: Extreme Value Performance Metrics; Figure S1: Cross-marking of clustering intervals.; Figure S2: NMR spectrum of Region 2 (−94.07~−46.65 ppm) with known functional groups and unknown structural fingerprints annotated.; Figure S3: NMR spectrum of Region 3 (−153.35~−105.93 ppm) with known functional groups and unknown structural fingerprints annotated.
