# Supplementary material for: Range-Aware Two-Stage Modeling for Feed Ratio Optimization in Fluoroelastomers: Mechanistic Pathways from NMR Structural Features to Macroscopic Properties
Source: Materials (Basel). 2025 Oct 6;18(19):4618. doi: 10.3390/ma18194618 (PMC12525985; doi:10.3390/ma18194618)
Supplement: Supplementary file 1 [file materials-18-04618-s001.zip › materials-3883997-supplementary.pdf]

Supplementary Information S1.1: NMR Data Preprocessing Parameters

Table S1- 1. Savitzky-Golay Filter Parameters

Parameter	Value	Rationale
Window size	11 points	Optimized for typical 19F-NMR peak width (6-10 ppm) in fluoroelastomers
Polynomial order	3	Balances noise reduction with peak shape preservation for −CF2− segments
Validation	Peak area retention >98%	Verified against standard fluoroelastomer samples

Table S1- 2. Baseline Correction (AsLS) Parameters

Parameter	Value	Reference/Justification
Smoothing parameter (λ)	10^5	Optimized for 19F-NMR baseline characteristics [1]
Asymmetric weighting	Applied to negative peaks	Accounts for upfield/downfield signal asymmetry
Quality criterion	Variation <0.5%	Industry standard for quantitative NMR analysis

Table S1- 3. Chemical Shift Calibration Standards

Reference Peak	δ (ppm)	Assignment	Literature
VDF main chain	-92 to -95	-CH2-CF2-	[[2],[3]]
TFE segment	-125 to -128	-CF2-CF2-	[[2],[4]]
PMVE side chain	-52 to -55	-OCF3	[[3],[4]]
Precision requirement	±0.02 ppm	Based on functional group separation analysis	

Table S1- 4. DBSCAN Clustering Parameters

Parameter	Value	Basis
Neighborhood radius (ε)	8 ppm	Statistical analysis of peak width distribution
Minimum samples	3	Noise filtering threshold (P<0.05 for random occurrence)
Cluster merging distance	2 ppm	Chemical shift measurement uncertainty
Final clusters	4 high-signal regions	Validated by expert spectroscopist review

Quality Control Thresholds:

- Peak area significance: >0.5% of total spectrum
- Signal-to-noise ratio: >3:1 for characteristic points

- Reproducibility: CV <5% for replicate measurements
- Final feature retention: 53 features (38 known + 15 unknown regions)

Supplementary Material S1.2: Extraction of Known Functional Group Features and Integral Area Calculation

1. Extraction of Known Functional Group Features

Based on long-term internal R&D experience, functional group information obtained from instrument software, and research results from polymer field core literature (e.g.,Polymer,Macromolecules) [[2]-[4]], nine chemical shift intervals and 33 characteristic points were systematically identified to construct a high-precision known functional group feature system. The chemical shifts, functional group assignments, and reference sources for each interval and characteristic point are detailed in **Table S1.2- 1** and **Table S1.2- 2**

Table S1.2- 1. Known Chemical Shift Ranges and Corresponding Functional Group Characteristics

Chemical Shift Range(ppm)	Corresponding Segment Structure	Attribution Monomer and Group
-52.9~-55.3	$-CF(CF_3O)CF_2-$	Perfluoromethylvinyl Ether (PMVE)-OCF_3
-50.3~-52.1	$-CF(CF_3O)CF_2-$	Perfluorooxyethylene (MO)-OCF_3
-70.8~-78.1	$-CF(CF_3)CF_2-$	Hexafluoropropylene (HFP)-CF_3
-57.6~-58.9	$-CF(CF_3O)CF_2-$	Perfluorooxyethylene (MO)-OCF_2O
92.5~94.4	$-CH_2(CF_2)CH_2-$	Vinylidene Fluoride (VDF)-CF_2
108.5~115.1	$-CF_2(CF_2)CH_2-$	VDF and HFP Copolymer Segment -CF_2
116.0~120.7	$-CF-CF_2(CF_2)CF_2-$	TFE and HFP Copolymer Segment -CF_2
121.1~125.6	$-O-CF-CF_2(CF_2)CF_2-$	TFE/PMVE/MO Terpolymer Segment -CF_2
125.7~128.4	$-CF_2(CF_2)CF_2-$	Main Chain of Tetrafluoroethylene (TFE) -CF_2

Table S1.2- 2. Known Chemical Shift Characteristic Points and Their Functional Group Assignments

Chemical shift	Functional group sequence
-146.59	-CH2-CF2-CF*-(OCF3)-CF2-CH2-
-145.95	-CF2-CF2-CF*-(OCF3)-CH2-CF2-
-145	$-CH_2-CF_2-[CF_2-CF(OCF_3)]-CF_2-CH_2-$
-128.05	-Rf-CF2-CF2-CF2*-CF2-Rf-
-126.91	-CF2-CF2-CF2-CF2*-CF2-Rf-
-126.8	$-[CF_2-CF(OCF_3)]-CH_2-CF_2-$
-126.32	-CH2-CF2-CF2-CF2*-CF2-CF2-
-124.13	-CF2-CF2-CF2-CF2*-CF2-CH2-CF2-
-123.91	-CF2-CF2-CF(OCF3)-CF2*-CF2-
-123.63	-CF2-CF(OCF3)-CF2*-CF2-
-123.4	-CH2-CF2-CF2*-CF2-CF(OCF3)-
-122.5	$-[CF_2-CF(OCF_3)]-$
-116.97	-CH2-CF2*-CF(OCF3)-CF2-
-115.7	$-(CH_2-CF_2)-(CF_2-CH_2)-(CH_2-CF_2)-$
-114.8	$-CH_2-CF_2H$
-114.67	-CF2-CH2-CF2*-CF2-Rf-
-113.7	$-(CH_2-CF_2)-(CF_2-CH_2)-(CH_2-CF_2)-$
-113.48	-CF2-CH2-CF2*-CF2-CH2-
-112.38	-Rf-CH2-CF2*-CF2-CH2-
-111	$-(CH_2-CF_2)-[CF_2-CF(OCF_3)]-$
-110.75	-CH2-CF2-CH2-CF2*-Rf-
-110.16	-Rf-CH2-CF2*-CF2-Rf-
-109	$-CF_2CH_2I$
-95.36	-Rf-CF2-CH2-CF2*-CH2-Rf-
-94.8	$-(CH_2-CF_2)-(CF_2-CH_2)-(CH_2-CF_2)-(CH_2-CF_2)-$
-94.21	-CF2-CH2-CF2*-CH2-Rf-
-92.59	-CF2-CH2-CF2*-CH2-CF2-
**Chemical shift**	**Functional group sequence**
-92	$-CF_2-CH_2-CF_2-CH_2-CF_2-$

-73	$-CF_2CF(OCF_3)I$
-59	$-CF(OCF_3)CF_2I$
-53.17	-CF2-CH2-CF(OCF3*)-CF2-CF2-
-52	$-OCF_3$
-40	$-CH_2CF_2I-$

2. Integral Area Calculation for Known Functional Groups

For the nine chemical shift intervals, the trapezoidal integration method was used to calculate peak areas, which were normalized to relative peak areas using the formula Eq(**Error! Reference source not found.**):

$$A_{rel} = \frac{\sum_{i=1}^{n} \frac{(I_i + I_{i+1})}{2} \times (\delta_{i+1} - \delta_i)}{A_{total}} \quad (1)$$

where A_{rel} is the relative peak area, I_i is the signal intensity of the i-th data point, δ_i is the corresponding chemical shift, and A_{total} is the total peak area of the entire spectrum. This method aligns with the spectral feature preprocessing standards recommended by Kalambet et al. [1], which demonstrates that the trapezoidal integration method can estimate peak areas with extremely high precision, achieving a 0.1% integration error level for Gaussian peaks with only 0.62 points per standard deviation.

For the 33 chemical shift characteristic points, the signal intensity at each point was extracted as a feature value and normalized to ensure data consistency and comparability. To ensure data quality, features with peak area proportions <0.5% of the total spectrum or excessively low signal intensities were excluded. Finally, 38 valid known features were retained for subsequent analysis.

Supplementary Material S1.3: Dynamic DBSCAN Clustering and NMR Spectrum Annotations for Known/Unknown Features

The following figures (**S1**–**S3**) integrate known functional group features (from **Table S1.2-1** and **Table S1.2- 2**) and unknown structural fingerprints (from DBSCAN clustering, **Table S1-4**) directly onto NMR spectra. Each annotation includes:

1.Feature name (e.g., F5, U12) for rapid cross-referencing with tables and text.

2.Functional group sequence (for known features) to clarify structural attribution.

1. Chemical Shift Interval Partitioning via DBSCAN Clustering

To address issues of broad peak overlap and complex signals in spectra, dynamic DBSCAN clustering was employed with a dynamic neighborhood radius set to 8 ppm, significantly reducing the over-segmentation rate. The minimum number of samples per cluster was set to 3 to avoid misclassifying noise points as valid signals. (The dynamic neighborhood radius of 8 ppm was determined based on spectral peak width statistics (average peak width 6-10 ppm), and the minimum sample number of 3 was used to filter instrument noise (single noise point continuous occurrence probability <5%).) Initial clustering results were analyzed for spatial overlap, and adjacent clusters with distances <2 ppm were merged, ultimately optimizing into four high-signal characteristic intervals. This provided effective structural fingerprint intervals for subsequent knowledge-embedded feature engineering.

2. Extraction of Unknown Structural Fingerprints

For the portions of high-signal regions identified by the clustering algorithm that were not covered by known features **(Figure S 1** shows cross-marking of known features and clustering intervals), non-overlapping division was performed at 0.2 ppm precision, with a dual screening criterion: excluding intervals with peak area proportions <0.1% of the total spectrum and intervals with <5 data points. Finally, 15 unknown features (U1-U15) were extracted. The dynamic DBSCAN clustering method used in this study breaks through the limitation of traditional methods that only focus on known functional groups. By systematically mining structural fingerprints in unknown regions, it improves the utilization efficiency of 19F-NMR spectral data. This excavation of unknown structural information not only enriches the feature space but also provides possibilities for discovering new structure-property correlations. These features, as unassigned "structural fingerprints," contain molecular structure information that has not yet been interpreted in their signal intensities and distribution characteristics.

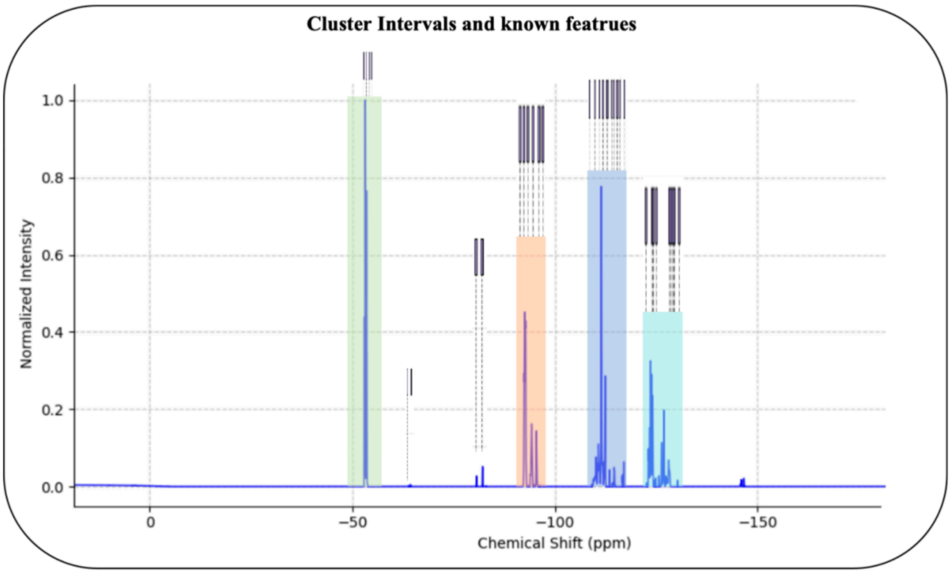

Figure S 1. Cross-marking of clustering intervals.

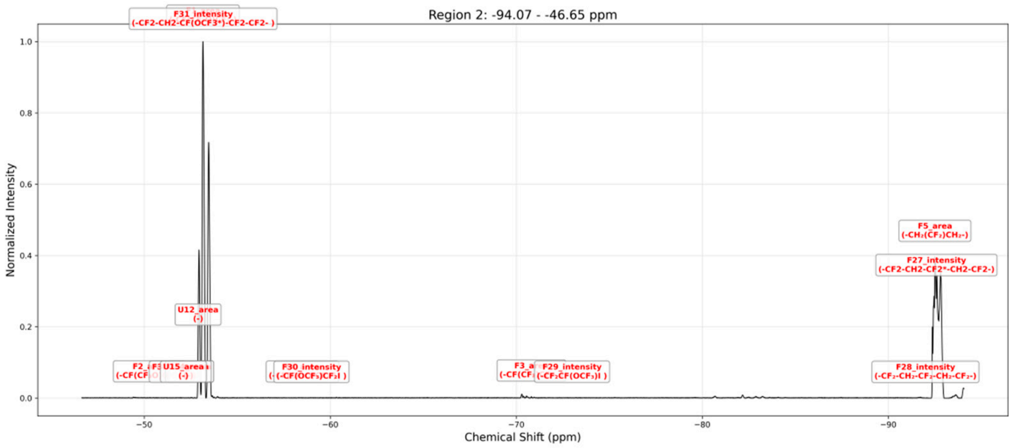

Figure S 2. NMR spectrum of Region 2 (-94.07~-46.65 ppm) with known functional groups and unknown structural fingerprints annotated.

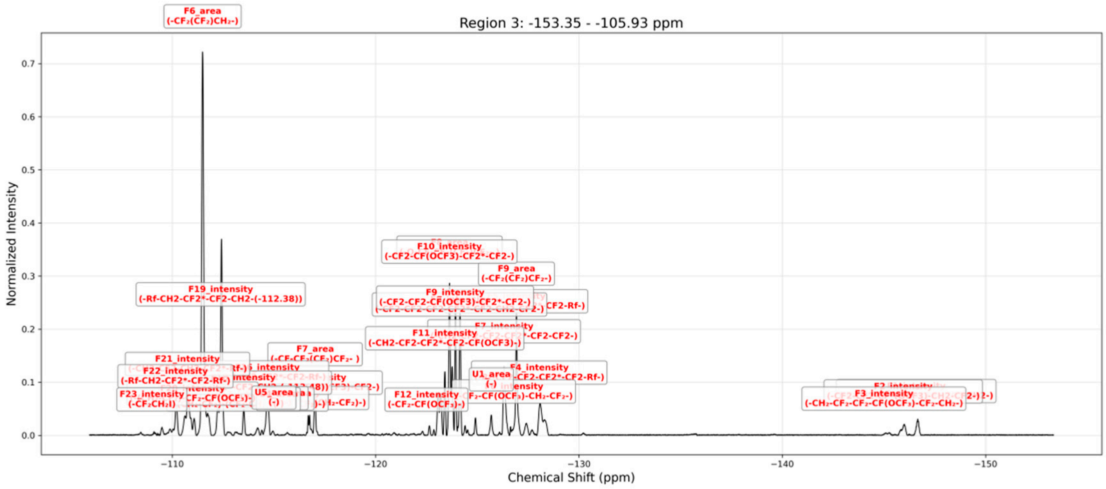

Figure S 3. NMR spectrum of Region 3 (-153.35~-105.93 ppm) with known functional groups and unknown structural fingerprints annotated.

Supplementary Material S1.4: Range-Aware Feature Engineering for Enhanced Extreme Value Sensitivity

S1.4.1 Range-Aware Scoring Methodology

Traditional feature selection methods based solely on linear correlation coefficients may inadequately capture the discrimination capability of features in extreme performance regions. This study implements a range-aware scoring approach that evaluates feature effectiveness by comparing global variance to within-group variance after quartile-based segmentation.

Table S1.4-1. Range Score Calculation Framework

Component	Definition	Mathematical Expression	Purpose
Global Standard Deviation	Population-wide target variability	σ_global = std(target_values)	Baseline variance measurement
Quartile Segmentation	Division into 4 equal groups	Q1, Q2, Q3, Q4 based on feature values	Create comparable subgroups
Group-wise Variance	Within-group target variability	σ_group_i = std(target_values_in_group_i)	Subgroup variance measurement
Range Score	Variance ratio metric	RS = σ_global / (mean(σ_group) + ε)	Feature discrimination ability

Score Interpretation:Higher range scores indicate superior ability to discriminate between different performance levels, particularly in extreme value regions.

S1.4.2 Functional Group Ratio Feature Construction

Based on key functional group patterns identified in NMR analysis, continuous ratio features are constructed to capture structural balance relationships:

Table S1.4-2. Key Functional Group Ratio Features

Ratio Feature	Construction	Structural Significance	Range Enhancement
OCF_3/CF_2-CF_2 Ratio	Σ(OCF_3_features) / Σ(CF_2CF_2_features)	Side-chain to backbone balance	Primary discrimination metric
PMVE/TFE Ratio	PMVE_ratio / TFE_ratio	Monomer composition balance	Secondary balance indicator
VDF/TFE Ratio	VDF_ratio / TFE_ratio	Main-chain composition ratio	Alternative composition metric
Group Relative Content	Group_sum / Total_signal	Normalized functional group abundance	Cross-sample normalization

S1.4.3 Threshold-Based Classification Indicators

To enhance extreme value sensitivity, continuous ratio features are transformed into binary classification indicators using data-driven thresholds:

Table S1.4-3. Range Indicator Construction Strategy

Indicator Type	Threshold Definition	Sample Classification	Extreme Value Enhancement
High-Value Indicator	Feature > 75th percentile	Top 25% samples	High-performance identification
Low-Value Indicator	Feature < 25th percentile	Bottom 25% samples	Low-performance identification
Medium-Value Indicator	25th ≤ Feature ≤ 75th percentile	Middle 50% samples	Balanced-state identification
Binary Encoding	0/1 classification	Discrete feature representation	Machine learning compatibility

Example Implementation:

- OCF_3_CF_2_high: Identifies samples with OCF_3/CF_2-CF_2 ratio above 75th percentile
- OCF_3_CF_2_low: Identifies samples with OCF_3/CF_2-CF_2 ratio below 25th percentile

S1.4.4 Extreme Value Sample Enhancement

Table S1.4-4. Extreme Sample Identification and Resampling

Parameter	Definition	Threshold	Enhancement Strategy
High Extreme	Upper performance boundary	$\mu + 0.8\sigma$	Performance enhancement target
Low Extreme	Lower performance boundary	$\mu - 0.8\sigma$	Performance optimization reference
Resampling Factor	Duplication multiplier	1.5×	Balanced enhancement intensity
Selection Criterion	Combined extreme identification	High OR Low extreme	Comprehensive extreme coverage

S1.4.5 Multi-Scale Feature Integration

Table S1.4-5. Feature Integration Hierarchy

Feature Level	Feature Type	Information Scale	Extreme Sensitivity
Level 1: Continuous Ratios	OCF_3/CF_2-CF_2, PMVE/TFE	Quantitative balance relationships	Moderate
Level 2: Range Indicators	_high, _low, _medium flags	Qualitative extreme identification	High
Level 3: Interaction Terms	Ratio × Ratio products	Multi-dimensional relationships	Enhanced
Level 4: Aggregated Groups	Functional group sums/means	Simplified structural representation	Moderate

S1.4.6 Feature Selection Weighting Strategy

Table S1.4-6. Composite Scoring System

Component	Weight	Calculation Method	Selection Priority
Range-Aware Score	70%	Quartile variance ratio	Primary discrimination
Correlation Coefficient	30%	Spearman rank correlation	Secondary relevance
Combined Score	100%	Weighted linear combination	Final ranking metric
Selection Threshold	Top-K	Configurable feature count	Typically 8-12 features

S1.4.7 Quality Control for Range-Aware Features

Table S1.4-7. Feature Quality Assurance Criteria

Quality Metric	Threshold	Action	Extreme Value Focus

Variance Threshold	$\sigma > 1\times10^{-5}$	Remove constant features	Preserve discriminative features
Range Score Minimum	RS > 1.0	Retain high-discrimination features	Prioritize extreme-sensitive features
Correlation Significance	$p < 0.10$	Statistical relevance filtering	Maintain predictive value
Extreme Sample Coverage	≥3 samples per extreme group	Ensure robust extreme representation	Validate extreme sensitivity

S1.4.8 Algorithm Configuration for Extreme Value Enhancement

Table S1.4-8. Implementation Parameters

**Parameter**	**Default Value**	**Optimization Range**	**Impact on Extreme Sensitivity**
Quartile Groups	4	3-5	Range score resolution
Extreme Threshold	0.8σ	0.5σ-1.0σ	Extreme sample definition strictness
Weight Ratio (Range:Corr)	70:30	60:40-80:20	Balance discrimination vs. relevance
Resampling Factor	1.5	1.0-2.0	Extreme sample enhancement intensity
Maximum Features	10	5-15	Model complexity vs. performance

S1.4.9 Validation of Extreme Value Sensitivity

Table S1.4-9. Extreme Value Performance Metrics

**Validation Metric**	**Calculation**	**Target Improvement**	**Interpretation**
Extreme Sample RMSE	RMSE(extreme_samples)	<15% of target range	Extreme prediction accuracy
High-Performance R^2	R^2(samples > 90th percentile)	>0.4	High-value sample modeling
Low-Performance R^2	R^2(samples < 10th percentile)	>0.3	Low-value sample modeling
Range Coverage	max(predicted) - min(predicted)	>80% of true range	Full range representation

This range-aware feature engineering approach specifically addresses the challenge of performance prediction in narrow-distribution datasets by constructing features that emphasize extreme value discrimination through functional group ratio analysis and threshold-based classification indicators.

References

[1] Kalambet Y, Kozmin Y, Samokhin A. Comparison of integration rules in the case of very narrow chromatographic peaks[J]. Chemometrics and Intelligent Laboratory Systems, 2018, 179: 22-30.

[2] Twum E B, McCord E F, Lyons D F, et l. Multidimensionl 19F NMR nlyses of Terpolymers from Vinylidene Fluoride (VDF) – Hexfluoropropylene (HFP) – Tetrfluoroethylene (TFE)[J]. Mcromolecules, 2015, 48(11): 3563-3576.

[3] Boyer C, meduri B, Hung M H. Telechelic diiodopoly (VDF-co-PMVE) copolymers by iodine trnsfer copolymeriztion of vinylidene fluoride (VDF) with perfluoromethyl vinyl ether (PMVE)[J]. Mcromolecules, 2010, 43(8): 3652-3663.

[4] d Cunh F R, Dvidovich I, Tlmon Y, et l. Emulsion copolymeriztion of vinylidene fluoride (VDF) with perfluoromethyl vinyl ether (PMVE)[J]. Polymer Chemistry, 2020, 11(13): 2430-2440.
